# Why do we study sphingolipids?

**DOI:** 10.1007/s00424-024-03020-0

**Published:** 2024-09-19

**Authors:** Anthony H. Futerman

**Affiliations:** Department of Biomolecular Sciences, Weizmann Institute of Science, 7610001 Rehovot, Israel

**Keywords:** Sphingolipids, Ceramide, Sphingosine 1-phosphate, Metabolic pathways, Evolution

## Abstract

Research on sphingolipids has proliferated exponentially over the past couple of decades, as exemplified in the findings reported at the International Leopoldina Symposium on Lipid Signaling held in Frankfurt in late 2023. Most researchers in the field study how sphingolipids function in regulating a variety of cellular processes and, in particular, how they are dysregulated in numerous human diseases; however, I now propose that we implement a more holistic research program in our study of sphingolipids, which embraces a sense of awe and wonder at the complexities and beauty of sphingolipids and of sphingolipid metabolism. I will outline the chemical complexity of sphingolipids, their modes of interaction within the lipid bilayer, and their biosynthetic pathways. I will then briefly touch upon the ability of current neo-Darwinian mechanisms to explain the emergence of both sphingolipids and of the complex pathways that generate them. Although such discussion is normally considered taboo in biological circles, I nevertheless submit that in-depth analysis of the minutiae of metabolic pathways, such as those of the sphingolipid biosynthetic pathway, raises challenges to current neo-Darwinian mechanisms that should not be shunned or ignored.

## What kind of questions do sphingolipid researchers normally ask?

For many of us, including Prof. Andrea Huwiler, whose untimely passing away we remember in this special issue [1], scientific research has played a major role in our lives. For most of the authors who have contributed to this volume, the focus of their scientific activities has been the study of sphingolipids, a unique class of membrane lipids that are involved in a plethora of cellular processes. We study *how* sphingolipids work, for instance, in health and disease [2]; we study *how* sphingolipid metabolism influences other biochemical pathways [3]; we study *how* sphingolipid metabolism itself is regulated [4]. That is to say, we study mechanism and *how* questions but tend not to study *why* questions.

Do *why* questions have a place in contemporary empirical science? A typical definition of science taken from the Oxford Dictionary defines science as “the systematic study of the structure and behavior of the physical and natural world through observation, experimentation and the testing of theories against the evidence obtained” or “knowledge or a system of knowledge covering general truths or the operation of general laws especially as obtained and tested through the scientific method” (Miriam-Webster dictionary). That is to say, the scientific method involves experimental testing of general laws, without addressing *why* there are laws in the first place. Is the metaphysical question of “why is there something rather than nothing” (or “why are there sphingolipids rather than no sphingolipids”) out-of-place in a scientific discussion, and does this kind of question rather belong to the realm of philosophy and metaphysics?

I presume that the majority of the authors of chapters in this special issue are actually Doctors of Philosophy, that is to say, they were awarded a PhD degree by one or other distinguished center of learning. Clearly, the award of a PhD degree (rather than, for instance, a Doctor of Science degree) to someone who has studied empirical science appears to be somewhat anachronistic, but nevertheless, it does tease the question of whether room should be found for a more philosophical approach when we carry out our scientific studies, and crucially, whether we should spend more time and energy considering the (philosophical) implications of our observations and scientific theories. Indeed, many a Nobel Laureate, after receiving this most distinguished of prizes, has metamorphosized into a student of philosophy^1^ and attempted to determine whether they can derive deeper (and existential) meaning from their life’s work. I would submit that such an approach should not be limited to Nobel Laureates in the twilight of their careers but is the prerogative of everyone who has spent days and nights in the laboratory as they struggled to meet the requirements of their PhD degree.

The award of a PhD degree itself does not turn sphingolipidologists into card-carrying philosophers [5] (although perhaps amateur philosophers!), but I would nevertheless submit that encouraging a more philosophical approach to our scientific studies would not be detrimental and might even be beneficial. Thus, it was during the Leopoldina Symposium on Lipid Signaling, held in Frankfurt in September 2023, that I first raised some of the issues mentioned herein with an audience of my sphingolipid peers (which included Prof. Huwiler). A significant amount of interest was shown in this approach, perhaps because the talk was somewhat unusual, at least compared to the other talks given at the symposium, but also possibly since some of the attendees were on board with the suggestion that room should be made for a more philosophical approach in our study of the natural sciences and in the conclusions we reach.

In the current short article, written to honor the memory of Prof. Huwiler, I will first ask the prosaic question of *why* we study sphingolipids. Having done so, I will then briefly discuss my personal perspective on the implications of these studies, including the philosophical ramifications as I see them. My conclusion, which the reader is free to agree or disagree with, is that not only can existential meaning be derived from our mechanistic studies of sphingolipids, but that such an approach enriches both our scientific research and our day-to-day outlook on life.

## Why do we study sphingolipids?

The reason that we chose a specific research field for advanced study is one of life’s genuine mysteries! In the case of Andrea Huwiler, an overview of her career [1] reveals the choices that took her from initial studies in pharmacy, on to the renowned lipid laboratory of Prof. Edward Dennis, and eventually to her well-known work on sphingosine 1-phosphate. In my case, my PhD degree work on glycosylphosphatidylinositol (GPI)-anchored proteins, largely on the synaptic enzyme, acetylcholinesterase [6], led to my interest in membrane lipids. I was accepted for a postdoctoral fellowship in the laboratory of Richard Pagano in the Carnegie Institution in Baltimore, who had done ground-breaking research on the intracellular transport of sphingolipids [7]. Subsequently, I established my own independent research laboratory in the Weizmann Institute, where the initial focus of research was the intracellular transport of sphingolipids in cultured neurons [8]. Thus, it is fair to say that the reason why I have been studying sphingolipids for the past 35 years or so was a combination of circumstantial events, rather than a calculated strategy focused towards defining a career trajectory, and I suspect this is the case for many.

The reason that many others study sphingolipids is based on the roles that sphingolipids play in human health and disease. The accumulation of sphingolipids due to mutations in the lysosomal enzymes that degrade them [9], such as in the inherited metabolic diseases, Gaucher disease, and Tay-Sachs disease, was a major historical motivation for studying sphingolipids. More recently, sphingolipids have been implicated in many other diseases, ranging from cancer to neurological to inflammatory diseases [9, 10]. Whether these diseases are caused by defective sphingolipid metabolism, or whether sphingolipids or defective sphingolipid metabolism play a downstream role in disease development and etiology is an ongoing debate, but irrespective of whether sphingolipids are the cause or a downstream response in the many diseases with which they are associated, this does not belittle the importance of studying how sphingolipids influence disease.^2^ Hence, funding for basic, applied, and medical research on sphingolipids has been readily available in the past couple of decades, with a number of companies also focusing on therapeutics directed towards intervening in the sphingolipid metabolic pathway. One of the more unexpected associations between sphingolipids and disease discovered in the past 15 years or so is the genetic connection between mutations in the *GBA* gene, the gene that encodes acid-beta-glucosidase, the lysosomal enzyme that cleaves the simple glycosphingolipid, glucosylceramide, and Parkinson’s disease [11]. It is now known that (heterozygous) mutations in *GBA* are the most common genetic risk factor for Parkinson’s disease [12], which has had a huge impact on the study of sphingolipid synthesis and degradation, and how the pathways of degradation are regulated.

While the reasons given above are entirely valid reasons for studying sphingolipids, I would like to suggest that another equally valid motive for studying sphingolipids, and the sphingolipid metabolic pathway, is a sense of curiosity and fascination with the world around us. Curiosity and the ability to analyze and verbalize questions might be considered one of the characteristics by which humankind differs from other members of the animal kingdom. And what could be more fascinating than a pathway which is regulated at multiple points and at multiple intracellular locations, a pathway that generates a number of different signaling molecules that are all metabolically interconnected, and a pathway that appears to regulate multiple cellular and extra-cellular processes. Moreover, I would also submit that such curiosity-driven research should imbue us with a sense of awe and wonder. I am not the first to suggest this; recently, a series of articles was published in the *Annals of the New York Academy of Sciences* [13, 14] which arrived at similar conclusions. To quote from one of the articles [13] in this special issue, “Scientists engage in their research for a variety of reasons—as diverse as their research interests are. But at the core, we find the same sense of awe and wonder that inspires spiritual ways to look at the world.” Would it be beyond the realms of rational thinking to suggest that just as we often stand in awe at the beauty of the natural world, we should likewise stand in awe and wonder [13] at the beauty [5] and complexity [15] of sphingolipids and of the metabolic pathways that generate and degrade them? To quote one of our recent papers [5], “while the notion of beauty is not generally considered a subject for debate or discussion in contemporary scientific literature…. we advocate that sphingolipids are not only enigmatic, as first suggested by Thudicum upon his discovery of Sphingosin in 1884, but also display inordinate beauty.”

With these thoughts in mind, I will now briefly discuss two aspects of sphingolipid biochemistry which produce in me a sense of awe and wonder, namely, their chemical structures and their modes of interactions with other components of the membrane lipid bilayer, and their metabolic interrelationships.

The reader will have to decide whether it is legitimate to attempt to derive existential meaning from such studies, or whether it is intellectually satisfying to simply asking *how* the pathway works.

## The chemical structure of sphingolipids and their modes of interaction with their environment

Unlike glycerolipids, which contain two hydrophobic acyl chains attached via an ester bond to the glycerol backbone, one of the two hydrophobic chains in sphingolipids is attached to the sphingolipid backbone (the sphingoid long chain base, (LCB)) via an amide bond. This is the result of the unique biosynthetic pathway (see next section) by which sphingolipids are generated in the endoplasmic reticulum (ER). An additional important chemical difference between sphingolipids and most glycerolipids is that sphingolipids have the ability to donate hydrogen bonds (from the OH moieties at the C1 and C3 positions of the sphingoid LCB and from the NH moiety at C2 to which the N-acyl chain is attached). Moreover, sphingolipids have a vast structural complexity, with the latest estimates from the Lipid Maps website (http://www.lipidmaps.org) documenting the existence of at least 1822 sphingolipid species (excluding glycosphingolipids), with computational analysis suggesting that up to 38,886 sphingolipid species could theoretically exist in nature based on the current known chemistry of sphingolipids.

The reason for this huge compositional complexity is the relatively large number of functional groups that can be modified, on both the sphingoid LCB and on the *N*-acylated fatty acid chain. Thus, the C1 position of the LCB can be modified by the addition of phosphorylcholine to form sphingomyelin, by the addition of phosphate to form ceramide 1-phosphate, or by the addition of simple and complex glycans to form glycosphingolipids; moreover, the C1 position can lack the hydroxyl moiety, generating the toxic, non-canonical LCB analogs, deoxy-SLs [16]. Essentially, each of the first five carbon atoms in the initial portion of the sphingoid LCB can be modified one way or another. For the *N*-acylated fatty acid, both the length and saturation status render a large number of possible chemical structures.

**Fig. 1:**
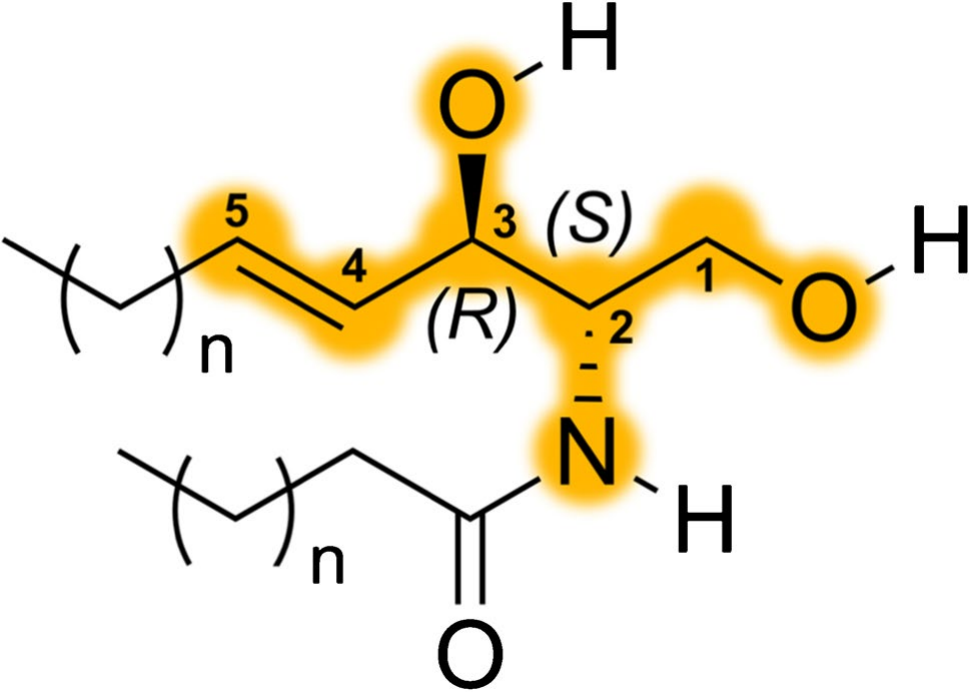
The sphingoid motif. Differences between the chemical structure of sphingolipids and of other membrane lipids are epitomized in the first 5 carbon atoms of the sphingoid long chain base (numbered), known as the sphingoid motif [5]. The sphingoid motif is generated from two carbon atoms (C1 and C2) supplied from an amino acid (normally serine) and from three carbon atoms (C3, C4, and C5) donated from a fatty acyl CoA, normally palmitoyl CoA. Modification of the sphingoid motif occurs by a series of enzymatic reactions which generate a fully functional sphingolipid

**Fig. 2:**
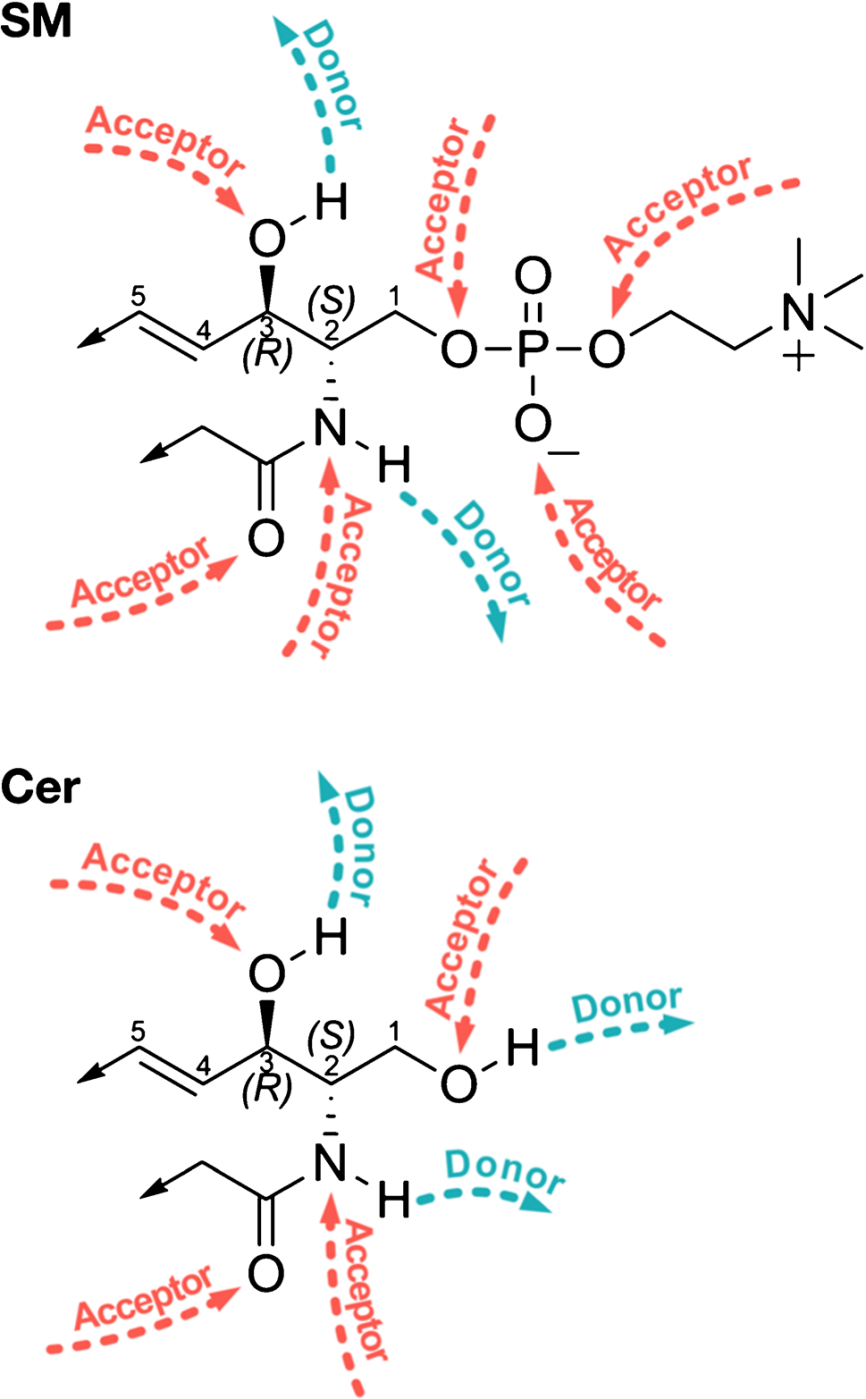
Hydrogen bond donors and acceptors in the sphingoid motif. The sphingoid motif, unlike most other membrane lipids, can both accept and donate hydrogen bonds. This expands, in principle, the number of interactions that sphingolipids can undergo in the membrane bilayer. Because of its phosphorylcholine head group, sphingomyelin (SM) can partake in more hydrogen bonds than ceramide (Cer). Using atomic level molecular dynamics simulations, we are currently quantifying some of these interactions with a view to understanding the role that these hydrogen bonds play in the fine-tuned structure of the membrane lipid bilayer

We recently suggested that these first 5 carbon atoms of the sphingoid LCB be known as the “sphingoid motif” [5] (Fig. 1), which is largely responsible for the unique chemical, biochemical, and biophysical properties of sphingolipids, permitting them to undergo a large number of interactions with other membrane components. Sphingolipids are perhaps most well-known for their ability to form membrane microdomains (often known as “rafts”), which are due to a slew of hydrophobic interactions on the acyl chain and due to hydrogen bond formation in the sphingoid motif (Fig. 2). The observation of large numbers of inter-molecular interactions within the plane of the membrane lipid bilayer has led to a change in our thinking about the structure of the lipid bilayer, such that the classical fluid-mosaic model has largely been superseded by a more refined model; we advocated that a more contemporary description of the lipid bilayer would be that the lipid bilayer is a “finely-tuned molecular assembly” [15].

This proposed terminology was chosen to better reflect lipid lateral heterogeneity within the plane of the lipid bilayer, lipid asymmetry across the bilayer, and specific protein-lipid and lipid-lipid interactions at the atomic level. The term “fine-tuning” is rarely used in biology but widely used in cosmology and physics [17]. Might one of the reasons that fine-tuning is used less in biology be due to its existential implications, i.e., why is life (or the universe) the way it is, rather than some other way, or how can the extraordinary low tolerance to changes in the physical and cosmological constants be explained? The magnitude of the tolerance to changes in the cosmological and physical constants is not necessarily reflected in biological systems inasmuch as biological systems, such as membrane lipid bilayers, show a higher tolerance to change and modification, whereas only minute changes can be tolerated in, for instance, the cosmological or the gravitational constants, or the universe would not exist in a form that supports life. Within cells, lipid bilayer composition changes along the secretory pathway, with the mole percent of SLs and cholesterol increasing from the ER to the plasma membrane [18]. Remarkably, the length and characteristics of the amino acids in the transmembrane domains of proteins that reside in these different lipid bilayers are perfectly matched to the width of the lipid bilayer (which is itself determined by the precise lipid composition within the bilayer [19]). Likewise, the helices of the transmembrane domains that span the bilayer are asymmetric in their surface area, matching the difference in lipid packing between leaflets [15]. Thus, even though lipid bilayers vary in their composition within cells, between organelles, and between organs (i.e., the lipid composition of the plasma membrane of a hepatocyte differs widely from that of a neuron), they appear to be fine-tuned in each specific case so as to carry out their functions and to allow atomic-level interactions with membrane proteins and other lipids.

An example of fine-tuning in the realm of sphingolipid biology is the interaction between ceramide, generated in the ER by the transmembrane proteins, ceramide synthases and sphingolipid delta (4)-desaturase (DES1), and the first enzyme in the biosynthetic pathway, namely, serine palmitoyl transferase (SPT). Thus, hydrogen-bonding and hydrophobic interactions formed between Asn13 and Phe63 in the SPT complex and ceramide (generated downstream) can influence the ceramide content of the ER. The highly specific interaction between ceramide and two amino acids in the SPT-ORMDL complex not only suggests finely-tuned molecular (atomic) interactions between sphingolipids and membrane proteins but also demonstrates a metabolic feedback loop which is crucial for the tight regulation of levels of SL biosynthesis. Together, the intricacies of the chemistry of the sphingoid motif, other moieties in the SL structure, and their modes of interaction with other membrane components not only inspire further study but leave us with a sense of awe and wonder.

## Sphingolipid metabolic pathways and the SL anteome

As mentioned above, sphingolipids are formed by a complex metabolic pathway. This is clearly not unique to SLs, as all cellular metabolites, by definition, are generated by complex metabolic pathways. What has been of some surprise to researchers in the sphingolipid field is the large number of ways in which the pathway is regulated, including metabolic feedback loops (see above). Of no less surprise were findings that many of the enzymes in the pathway are found as multiple isoforms, which is perhaps best exemplified by the ceramide synthases, of which six isoforms exist in mammals [20]. The main difference between these isoforms is their ability to use fatty acyl CoAs of different chain lengths to generate (dihydro)ceramides with specific *N*-acyl chain lengths. This implies that the sphingolipid *N*-acyl chain length is of great importance for cell physiology, a suggestion supported by the changes observed in the *N*-acyl chain length in a number of human diseases [21], and the effect of the *N*-acyl chain length on membrane biophysical properties [22].

**Fig. 3:**
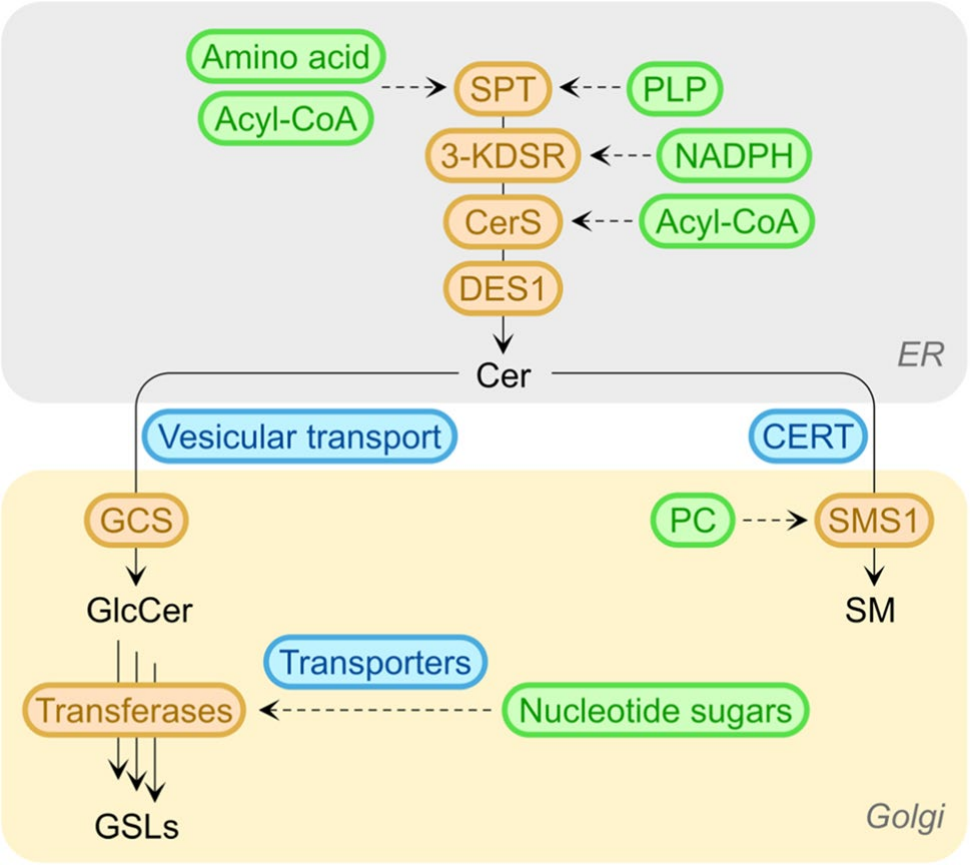
A simplified scheme of the anteome of the pathway of sphingolipid biosynthesis. Recently, we introduced the concept of the anteome, i.e., those pathways which impinge upon the pathway of sphingolipid synthesis (or of any other metabolic pathway) which are essential for the functioning of the pathway [4, 23, 24]. The scheme below shows the sphingolipid biosynthetic pathway, with the enzymes in orange, anteome components in green (note that each of the metabolites in the anteome are themselves generated by a complex metabolic pathway, which also has its own anteome), and accessory proteins and molecular machineries in blue. SPT, serine palmitoyltransferase; PLP, pyridoxal-5-phosphate; 3-KDSR, 3-ketodihydrosphinganine reductase; CerS, (dihydro)ceramide synthase; DES1, sphingolipid delta(4)-desaturase; SMS 1, sphingomyelin synthase 1; GCS, ceramide glucosyltransferase; CERT, ceramide transport protein; GSLs, glycosphingolipids; PC, phosphatidylcholine

Adding to the complexity and modes of regulation of the sphingolipid biosynthetic pathway are the metabolic components which feed into the pathway to allow it to function. Thus, we coined the term “anteome” to describe the network of metabolic pathways that are absolutely required for sphingolipid biosynthesis (Fig. 3). By way of example, SPT requires pyridoxal-5-phosphate (PLP), amino acids (normally serine but alanine and glycine can also be used) along with acyl CoA [4], whereas the generation of a glycosphingolipid in the Golgi apparatus requires a mechanism to transport ceramide from the ER to the Golgi apparatus [23], the generation of sugar nucleotides in the cytosol, followed by their transport into the lumen of the Golgi apparatus via sugar nucleotide transporters [24]. This being the case, there may be as many as five pathways consisting of ~ 28 enzymes and ~ 40 metabolites that are absolutely required for sphingolipid biosynthesis in the ER [4], not counting the molecular machinery required for ceramide transport and the multiple enzymes and pathways needed for glycosphingolipid biosynthesis in the Golgi apparatus.

The recognition of the two features mentioned above, namely, the finely-tuned interactions in which SLs are involved within lipid bilayers (Fig. 2) and the dependence of the SL biosynthetic pathway on anteome components (some of which are metabolites required by the pathway, and some are which are complex molecular machineries required for aspects such as ceramide transport between the ER and the Golgi apparatus, Fig. 3), inevitably leads to discussion of whether there are satisfactory evolutionary models that can explain the emergence of such a complex metabolic pathway. We fully appreciate that this question is more than a little contentious and often leads to heated debate in which sides are often taken, not necessarily based on the quality of the scientific evidence but rather on the philosophical implications of suggesting that the classical neo-Darwinian pathway may not provide quite as many clearcut answers as often assumed. In the case of the sphingolipid biosynthetic pathway (and surely in the case of other metabolic pathways), one major challenge is “what came first” (the chicken or the egg, or the Sphinx and the egg [24]?). Very few studies have addressed this issue in evolutionary biology, and many of the assumptions that are often put forward lack detail. As we learn more about the sphingolipid biosynthetic pathway, and as our understanding of the details of the pathway has evolved over the past 30 years or so, is it not legitimate to challenge whether the latest advances have altered our view of how the pathway may itself have evolved [23]?

## Are sphingolipids chemical scum?

I advocate that the view of beauty, awe, and wonder considered above is not only somewhat rare in the world of sphingolipid research but is likely not accepted by many active research scientists. Perhaps the most well-known of these is the physicist, the late Prof. Stephen Hawking, who suggested in an interview in 1995 that “the human race is just a chemical scum on a moderate-sized planet, orbiting around a very average star in the outer suburb of one among a hundred billion galaxies.” According to this reasoning, sphingolipids are also chemical scum. The exquisitely-regulated pathway of biosynthesis, involving multiple enzymes, each with its unique mode of regulation that depends largely on their three-dimensional structures, which itself is determined by sequence information in the genome, is chemical scum. The anteome, which provides essential metabolites, is chemical scum. The generation of the 3-OH moiety of the sphingoid long chain base, which acts to donate hydrogen bonds to other membrane components, is chemical scum. Sphingosine 1-phosphate, which Andrea Huwiler worked on for so many years, is chemical scum. Personally, I do not know anyone who has been in awe, or stood in wonder at the sight of chemical scum, and I would like to humbly suggest that sphingolipids are most definitely not chemical scum. Likewise, Prof. Hawking’s suggestion that human beings are chemical scum not only erodes human dignity but overlooks the fact that the ability of human scum to study and appreciate sphingolipid scum seems rather unlikely.

## Concluding remarks

**Fig. 4:**
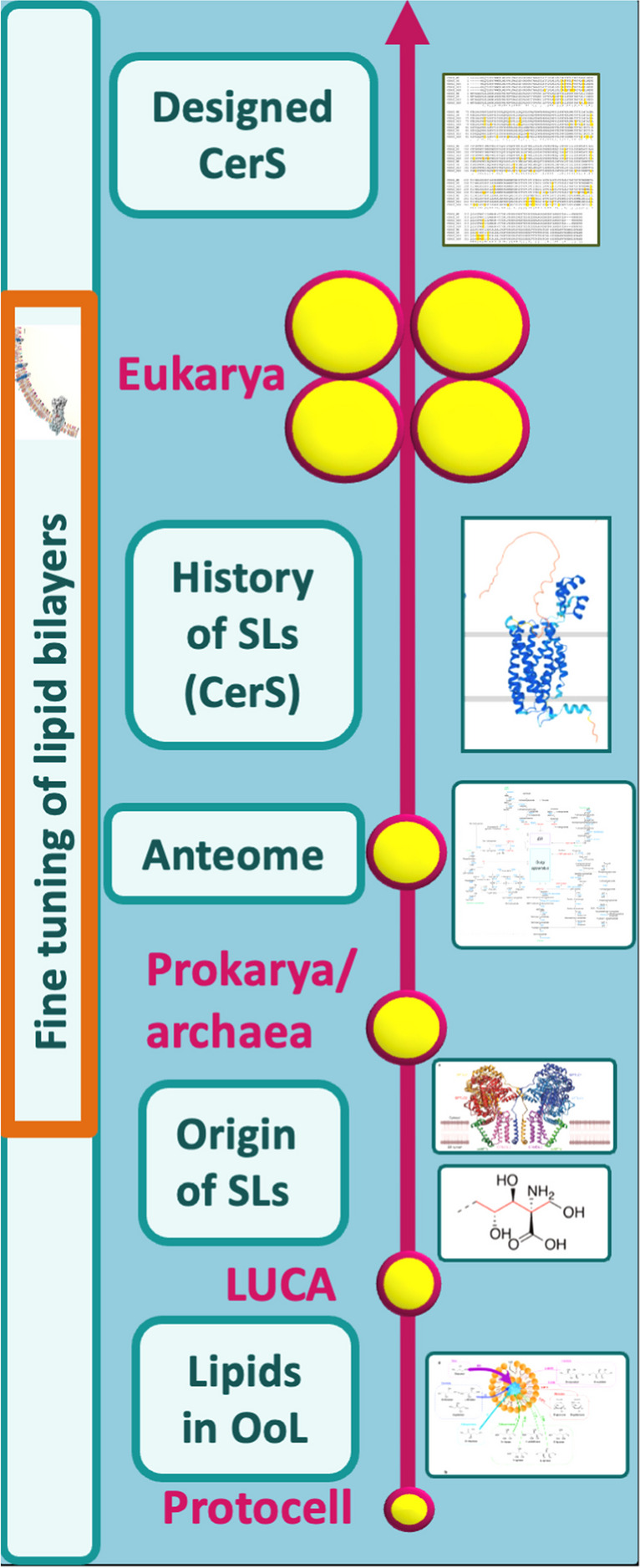
Schematic illustrating current research directions to examine the history of SLs and how the pathway may have emerged. The research program of the Futerman laboratory is currently focusing on the possible roles of (sphingo)lipids in the origin of life [25], determining where sphingolipids came from [5], the history of the emergence of the sphingolipid metabolic pathway along with its anteome [4, 23, 24], and whether ceramide synthases with enhanced stability and activity can be intelligently designed by computational methods [26]. Additional studies are focusing on how sphingolipids interact with other membrane components to help form the finely-tuned membrane lipid bilayer [15, 27]

I have mixed feelings in writing this short review. First, one of sadness for the loss of an esteemed colleague, but second, a sense of satisfaction that those of us left behind can continue the legacy of the work in which Andrea played such an important part. Whether the kind of existential and philosophical questions raised in this article find a place for discussion among the sphingolipid community will be determined as time goes on; this author would hope that the sphingolipid community expands its horizons to focus not just on mechanistic questions but to learn from distinguished colleagues (see footnote 1) who have attempted to look beyond their day-to-day research work and take advantage of their degrees in philosophy. With this in mind, the research program that we have recently initiated on fine-tuning of sphingolipid interactions and on the role of the sphingolipid anteome (Fig. 4) has inspired us to re-evaluate the currently accepted neo-Darwinian narrative to test whether it can explain the emergence of such a complex, multifaceted, beautiful, and awesome pathway.

## Data Availability

No datasets were generated or analysed during the current study.

## Footnotes

1 There are numerous examples, such as the late Stephen Weinberg, whose work on theoretical physics led to his award of the Nobel prize and who waxed philosophical in later life. Ditto Francis Crick, who expressed strong views on philosophy and religion, and John Goodenough, who received the Nobel prize in chemistry in 2019 and came to quite different conclusions from Weinberg and Crick. What unites these giants of their fields was their willingness to engage in thinking about existential questions (i.e., the meaning of life) and to think about the philosophical ramifications of their basic research findings.

2 The author of this article, along with the two editors of this special edition, organized a series of conferences on the ‘Molecular Medicine of Sphingolipids’ from 2012–2018 (see, for instance, https://conferences.weizmann.ac.il/MMS2018/molecular-medicine-sphingolipids).
